# Full Hierarchic Versus Non-Hierarchic Classification Approaches for Mapping Sealed Surfaces at the Rural-Urban Fringe Using High-Resolution Satellite Data

**DOI:** 10.3390/s90100022

**Published:** 2009-01-05

**Authors:** Tim De Roeck, Tim Van de Voorde, Frank Canters

**Affiliations:** Vrije Universiteit Brussel, Cartography and GIS Research Unit, Department of Geography, Brussels, Belgium; E-mails: tim.de.roeck@vub.ac.be (T. De R.); tvdvoord@vub.ac.be (T. Van de V.)

**Keywords:** Urban mapping, sealed surfaces, hierarchic classification, multiple layer perceptron, decision trees

## Abstract

Since 2008 more than half of the world population is living in cities and urban sprawl is continuing. Because of these developments, the mapping and monitoring of urban environments and their surroundings is becoming increasingly important. In this study two object-oriented approaches for high-resolution mapping of sealed surfaces are compared: a standard non-hierarchic approach and a full hierarchic approach using both multi-layer perceptrons and decision trees as learning algorithms. Both methods outperform the standard nearest neighbour classifier, which is used as a benchmark scenario. For the multi-layer perceptron approach, applying a hierarchic classification strategy substantially increases the accuracy of the classification. For the decision tree approach a one-against-all hierarchic classification strategy does not lead to an improvement of classification accuracy compared to the standard all-against-all approach. Best results are obtained with the hierarchic multi-layer perceptron classification strategy, producing a kappa value of 0.77. A simple shadow reclassification procedure based on characteristics of neighbouring objects further increases the kappa value to 0.84.

## 1. Introduction

Since 2008 more than half of the world population – 3.3 billion people – is living in cities. By 2030 this number will have increased to almost 5 billion [1]. In Europe, about 75% of the population now lives in urban areas and more than a quarter of the European Union's surface is covered by urban built-up areas. Not only because of the population increase, but also because of the changing lifestyle, the built-up areas are growing rapidly. People tend to use more space and that is why, in the last 20 years, while the population only increased by 6%, the built-up area increased by 20%. This urban sprawl is mainly occurring in regions with a high population density and economic activity [2].

One of the most obvious physical evidences of urban growth is an increase in the spread of sealed surface types [3]. Sealed surface distribution is therefore an interesting indicator for monitoring urban sprawl and its impact on the environment [4]. Recently, many studies have focused on the use of satellite imagery for mapping sealed surfaces [5]. Satellite images are indeed an interesting data source for monitoring urban dynamics. They have a high repetition cycle, which offers the opportunity to build up time series for change analysis. Furthermore, the spectral information present in satellite images can be used to automate the image interpretation process and to develop indicators describing various characteristics of urban development. In comparison with visual interpretation, which is still the standard approach in many operational applications, automated image interpretation is also less time and labour consuming.

In the past decade various new satellite platforms have been launched carrying multispectral sensors with high spatial resolution like Ikonos and Quickbird. The improvement in spatial resolution compared to traditional sensors like Landsat ETM+ or SPOT-HRV has substantially increased the potential of satellite remote sensing for urban applications. However, opposed to the advantages of the higher level of spatial detail offered by sensors like Ikonos and Quickbird, the interpretation of imagery produces by these sensors poses new challenges, particularly in urban areas. Because of the high spatial resolution of the images, pixels are mostly smaller than the urban objects that need to be distinguished. Hence, in most cases, one high resolution pixel does not contain information referring to the object as a whole, yet only to a specific material that is part of the object. The use of different building materials, the age of these materials, as well as differences in orientation and illumination result in a higher spectral heterogeneity within classes. Another limitation of high resolution optical imagery is the low spectral resolution. The small number of spectral bands that are available – usually blue, green, red and near infrared – obstructs the separation of classes solely on the basis of spectral information. Class heterogeneity and spectral confusion among classes also hamper the use of high-resolution optical sensors for sealed surface mapping. Previous research pointed out that the heterogeneity of sealed surfaces causes lots of confusion with bare soil [6]. Also dark building materials, shadow and water are often difficult to distinguish [7-9].

The consequence of the above is that traditional, pixel-based spectral classification methods cannot accurately map land cover in general, and sealed surfaces in particular, in complex settings like urban areas. The input of the classifier being spatially limited to a single pixel hampers the interpretation process. Context-based approaches, taking not only the spectral but also the spatial characteristics of the scene into account, may contribute to a better separation of spectrally similar surface types, and may therefore also be useful for high-resolution mapping of sealed surfaces.

### 1.1 Context-based approaches

Various post-classification approaches have been suggested to take image context into account and have been applied for urban mapping purposes, ranging from simple majority filters [10] or kernel-based reclassification [11] to structural pattern recognition techniques [12] or the use of context-based rules [6,9]. As an alternative for the post-classification approach, information about the surroundings of a pixel can also be used in the classification process itself. Several techniques like the use of the grey level co-occurrence matrix [13-14], the use of edge-based measures as extra input in the classification [15-16], texture spectrum encoding methods [17], context-related aggregation schemes based on the Dempster-Shafer theory of evidence [18] and the use of geometric activity features [19] have been proposed. The inclusion of context in a pixel-based classification approach though, requires an arbitrary setting of the window size within which texture or context measures are calculated. An inappropriate window size can substantially reduce the classification accuracy [14]. While multi-resolution approaches have been proposed to deal with issues of variable scale and support [14, 19-22], the fact that a regular window does not coincide with the actual boundaries of meaningful objects in the imagery remains a major drawback of the traditional, pixel-based classification approach, particularly in spatially complex scenes. It explains why in recent years object-based classification approaches have gained more attention, especially for the mapping of urban areas.

### 1.2 Object-oriented approach

In the object-oriented approach, the field of view for calculating texture or context information is not a user-defined, artificial window. Instead, the image is divided into objects based on a spectral homogeneity criterion. At the object level, geometric, texture and context variables can be defined. Together with the spectral information, these variables can then be used as input for the classification process. Because of the use of additional, object-specific non-spectral information, the object-oriented approach is promising for the classification of high-resolution scenes that are characterized by a strong within-class spectral heterogeneity and spectral confusion between classes [23-26]. Different studies have shown that an object-based classification approach can substantially improve classification results in comparison with pixel-based classification [27-30].

For the segmentation step in the object-oriented approach different methods have been proposed [31-35]. Ideally the objects resulting from the segmentation process should correspond to real-world objects. Over-segmentation occurs when one real-world object contains several image segments, while under-segmentation means that one image segment encloses different real-world objects. While over-segmentation can be made up for in the ensuing classification step, this is not the case for under-segmentation. Because one segment covers different real-world objects, the classifier used in the ensuing classification step will be unable to assign one correct target class for the entire segment. At least a part of the segment will be assigned wrongly. Under-segmentation thus directly results in classification errors and must therefore be avoided. This stresses the importance of a proper choice of the segmentation level. This choice is not always straightforward. Using the eCognition® software for segmenting high resolution remote sensing data, [36] reported irregular boundaries and the occurrence of faulty segments in areas of low contrast. Depending on the heterogeneity tolerance, [37] obtained separate image segments defining the transition zone between two real-world objects instead of sharply delineating the border between the two. The multiple pass segmentation algorithm used by [38] showed difficulties in clearly separating sealed surfaces covered by shadow and trees. [39] reported the occurrence of segments containing both sealed surfaces and lawns in segmentation of high resolution satellite images of urban areas. No automated methods are currently available to successfully select an optimal segmentation level for urban scene analysis [40-41]. The choice is therefore mostly left to the user who, through an iterative process of modification of segmentation parameters and visual inspection of the segmentation result, seeks for an optimal solution [24].

### 1.3 Non-parametric classification

Once an optimal segmentation is obtained and image segments have been described by a set of object-based features, the segments can be classified. Because constraints on class distributions imposed by parametric classifiers are seldom satisfied for non-spectral features, non-parametric classification algorithms are preferred. Very often a nearest neighbour classifier is applied [30, 42]. Of the full range of non-parametric classification methods though machine learning approaches like decision trees and neural networks tend to produce higher accuracies than the more traditional classifiers, especially in complex landscapes [43-46]. In contrast with the nearest neighbour classifier, these more sophisticated learning algorithms take into account the full spectral distribution of the training data.

A decision tree is a follow-up of numerous splits, each consisting of a binary decision. The input data is passed through the branches, ending in a leaf which represents one of the target classes. Each split corresponds with a well formulated rule for separating the training data that meets the formulated condition from the rest of the training data. Because of their intuitive simplicity, decision trees are easy to interpret by the user. Several tree construction approaches have been developed [47-49]. In recent years, decision tree algorithms have been increasingly used for object based land-use/land-cover classification of high-resolution satellite scenes of urban areas [7, 19, 50].

Artificial neural networks are inspired on human interpretation capabilities and attempt to simulate the complex processes in our brain. Relevant relations between the input data and the expected classification output are extracted without the necessity of putting them in strict rules. This makes the process a sort of black box, in the sense that the decision mechanism is not known. Numerous types of network architectures have been used for image classification, including radial basis function neural networks [51], ARTMAP [52-54], learning vector quantization [55] and self-organising maps [20, 56]. The most widely used artificial neural network paradigm for image classification is the multi-layer perceptron, using the backpropagation learning algorithm [57]. A multi-layer perceptron neural network (MLP) consists of several layers of nodes. In the training process these nodes ‘learn’ the relationship between input data and output classes. The resulting knowledge is then used to classify the image by processing the input data through the network. MLP have been increasingly used for land-use/land-cover classification of urban areas [9, 19, 45, 58-59].

While recently much work has been done on object-based land-cover classification of urban areas using high-resolution satellite data, only few studies have compared the performance of different types of classifiers or different classification strategies on the same data set. Reference [19] compares the performance of decision trees and multi-layer perceptrons for classifying man-made objects in a semi-urban environment, using window-based as well as object-based classification features, separately and in combination. Slightly better results are obtained with multi-layer perceptrons than with decision trees in all classification scenarios tested. Reference [50] compares different decision tree classification strategies for object-oriented mapping of urban land use from Ikonos imagery. The results of this study suggest that a one-against-all classification procedure carried out in successive steps, each time separating one class from the remaining set of observations and starting with the classes that are most easy to separate, gives more accurate results than a procedure in which the classification rules for separating all classes are acquired in one step.

### 1.4 Objectives of the study

The major objective of the research reported was to define an accurate method for the mapping of sealed surfaces in the rural-urban fringe, using high-resolution satellite imagery. Two different object-oriented classification scenarios were tested on an Ikonos image of an urban fringe area, covering part of the city of Ghent, Belgium, using both multi-layer perceptron (MLP) and decision tree (DT) learning algorithms, for distinguishing between six major target classes. In the first scenario, which we might refer to as the standard approach, one neural network (or one decision tree) was built to assign image objects to the different land-cover classes to be distinguished. In the second scenario the potential of a full hierarchic classification strategy was explored. In this approach the land-cover classification model was not built in one step, yet a series of classification models was defined to successively separate each land-cover class from the remaining classes. In both approaches, use was made of a set of 29 spectral and textural features available in Definiens®. For comparison reasons, a benchmark scenario using a nearest neighbour classification was also applied, using only the 4 multispectral bands and the standard deviations on these bands as input. An accuracy comparison of the different classification scenarios was carried out using a validation data set, consisting of three morphologically distinct test sites. The validation data was obtained by an exhaustive visual image interpretation of a high-density residential area, a low density built-up area and a rural zone.

Particular attention was paid to the presence of shadow in the imagery. By concealing the underlying ground cover, shadows cause a substantial loss of information. Especially in urban areas, where there are pronounced changes in surface elevation, shadow effects may be significant. Dealing with the presence of shadows can be subdivided into two sub-problems discussed by [8]: shadow detection and shadow removal. Even though shadows fall on different land-cover types, they have a distinct spectral signature and may be separated from other classes in the classification phase [60-61]. To avoid that shadow areas are wrongly attributed to other dark matter, such as water or dark artificial surfaces, a commonly used approach is to add shadow as a separate class in the classification [62]. Shadow areas can then be re-assigned to one of the target land-cover classes after the initial classification is obtained by defining a post-classification shadow re-assignment procedure. This can be achieved by applying context-based rules [63] or by performing a new classification [9]. The latter approach, with relative border to other classes as input, was followed in this study.

## 2. Study area and data

**Figure 1.**
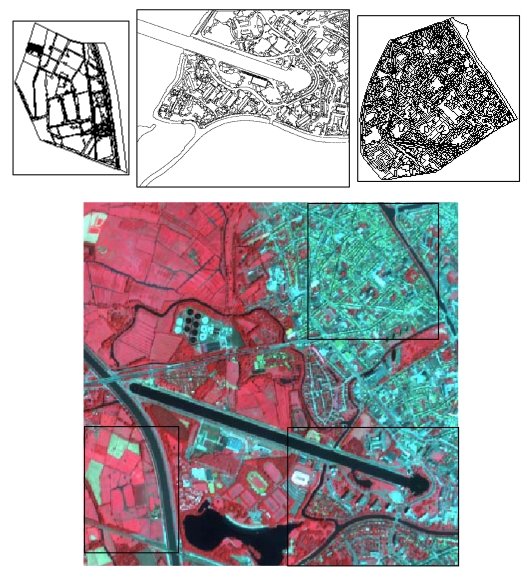
False colour infrared image of the study area (bottom) showing the spatial extent of the three ground truth sites (top).

The study area is situated in the southwestern part of the city of Ghent. It is covered by a square subset of an Ikonos image (Figure 1, bottom) acquired on August 5, 2003. The image subset consists of high-density residential areas in the northeastern part, more open, low-density built-up areas in the southeastern part and rural areas in the western part. The panchromatic image band has a spatial resolution of 1 metre and the four multispectral bands have a resolution of 4 metres. Through PCA-based pan sharpening the resolution of the multispectral bands was increased to 1 metre.

A 1:12,000 large-scale aerial photo mosaic from 2002 with a resolution of 25 cm covering the Ghent study area was made available by the Agency for Geographical Information of Flanders (AGIV). The aerial photographs were used for facilitating the visual collection of training and validation data from the Ikonos image.

## 3. Methods

### 3.1. Classification scheme, selection of training data and strategy for accuracy assessment

For producing a land-cover map of the study area separating sealed surfaces from other cover types, we distinguished six classes. Urban areas are mainly covered by sealed surfaces. Because of the characteristics of the study area with its many red roofs the sealed surface class can, both visually and spectrally, be divided into two different subclasses: a class consisting of red roofs, tennis courts with red gravel, running tracks and other red surfaces, and a class encompassing all other man-made surfaces like asphalt, concrete, etc. that mostly have grey tints. Other classes that were distinguished are water, vegetation, bare soil, and an additional shadow class. To be able to cover the full range of spectral heterogeneity of the grey and the red classes, we performed an initial unsupervised clustering to select training samples representing the different characteristics of both sealed surface classes. The final training set consisted of 150 training samples for each of the six classes, well distributed over the entire study area.

A first accuracy assessment of the performance of the different classification scenarios was carried out based on a set of randomly selected point locations. This traditional point-based validation approach, however, was not able to reveal the clear differences in the outcome of the various object-oriented classification strategies. We therefore produced an extensive ground truth data set by visually interpreting three morphologically distinct test sites with a different degree of urban density: a high-density built-up urban area, a less densely built-up area at the urban fringe and a rural site. A total of 4990 polygons were digitized on the Ikonos image. Figure 1 (top) shows the digitized polygons for the three sites. For each polygon in the visual interpretation covered by shadow the underlying land-cover type was registered as well. This information was used to assess the accuracy of the post-classification shadow re-assignment procedure. In order to minimize boundary effects caused by uncertainty in visual image interpretation, boundary pixels in the rasterized version of the polygon map were masked out through a buffer operation and were not considered in the validation process. Table 1 lists the total number of validation pixels for each target class.

### 3.2. Segmentation

**Figure 2.**
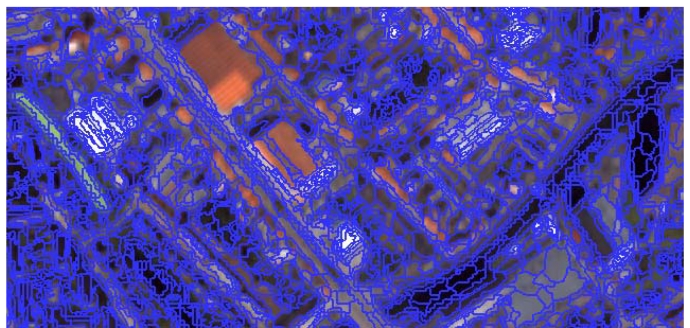
Segmentation of non-red surfaces for a subset of the study area (step two of the segmentation process).

The first step in an object-based classification approach is image segmentation. Depending on the spectral input and the segmentation method used, the defined image objects will be different. Several segmentation methods have been proposed, but in general the region-growing technique tends to give the better results [37, 64-65]. In this study we used the region-growing segmentation algorithm implemented in Definiens®. The method iteratively groups pixels until a predefined heterogeneity threshold is exceeded [66]. Different combinations of input variables and parameter settings were tested to optimally delineate the object borders between different classes. A combination of the four multispectral bands and the NDVI as input variables resulted in a satisfying segmentation result for five out of the six classes we attempted to classify. The contours of red objects, however, appeared to be badly outlined or not to show up at all. A segmentation approach based on the ratio between the green and the red spectral band allowed for a better outlining of the red surfaces present in the imagery. This segmentation result, however, was far from optimal for delineating the other classes. Therefore the segmentation was performed in two steps. First, a segmentation based on the ratio between the green and the red spectral band was produced. Using the resulting segments, red surfaces were separated from the other classes by applying one of the classification models (see below). To discriminate the other classes, a second segmentation (Figure 2) was produced for the remaining area, not classified as red surfaces, based on the four multispectral bands and the NDVI. The image objects obtained in the second segmentation step were assigned to one of the five remaining classes applying five different classification scenarios.

We deliberately opted for a relatively low heterogeneity threshold to ensure that boundaries between the six classes were properly identified. As can be seen from Figure 2, this leads to over-segmentation and automatically limits the potential of using size, shape and neighbourhood information for classification. However, since we were interested in identifying broadly defined land-cover types, rather than urban objects, like houses, parking lots, roads, etc. we did not expect to gain much from size/shape related information for distinguishing between these land-cover types. By increasing the heterogeneity threshold we observed that the boundary between different types of land cover, as we visually observed it, was less well delineated by the segment structure. That is why we chose for a relatively low scale parameter.

### 3.3. Classification algorithms

As a benchmark scenario, a standard nearest neighbour classification was performed, using the 4 multispectral bands and the standard deviations on these bands as input. A nearest neighbour classifier though, does not take into account the full spectral distribution of the training data. This may hamper classification in a complex urban or rural-urban setting, where image objects belonging to the same class often have rather different properties. Especially classes like bare soil and sealed surfaces may have rather heterogeneous spectral as well as textural characteristics [14]. We therefore applied two more sophisticated non-parametric classification algorithms that do take into account the full spectral distribution of the training data: decision trees (DT) and multi-layer perceptrons (MLP).

DT are often used for their simplicity and because the decisions made by the classifier in the classification process are easy to interpret. Their performance was compared with MLP which have shown to produce good results for classifying complex settings like urban areas [9, 19, 45, 59]. Classification results obtained with both classifiers were compared with the benchmark nearest neighbour classification. In this research we used the See5.0® software [49] for DT classification, NeuralWorks Predict® v3.12 for MLP classification and the Definiens® software for the nearest neighbour classification.

### 3.4. Feature selection

An object-oriented classification offers the opportunity to include, besides spectral information, also texture, size, shape and neighbourhood features as input for the classification process. Depending on the target classes, an optimal set of input variables needs to be selected from this range of possible input features. As explained earlier, the deliberate choice for a relatively low heterogeneity threshold, to ensure that boundaries between the six classes are properly identified, limits the potential of size, shape and neighbourhood information. Hence only spectral and textural features were considered as possible input variables for the classification. A set of 29 features available in Definiens® was selected for use in the classification process (Table 2).

From the set of possible input features, an optimal subset of features was selected for each step in the DT and MLP classification scenarios. For DT as well as MLP we made use of the feature selection algorithms embedded in the software: the ‘winnow’ function for DT in See5.0® and the genetic algorithm for MLP in NeuralWorks Predict®. For building MLP models, we first trained a network making use of the variable selection option in Predict. Afterwards a new MLP was trained, using only the previously selected variables as input features. To avoid overfitting of the MLP to the specific characteristics of the training set, the set was split up in a training subset and a test subset. While the training subset is used for the actual training of the model, the test subset is used for interrupting the training process before overfitting occurs. The ratio between training and test data was set to 60/40. For DT construction, besides the ‘winnow’ option, also the boosting function was used. Using this function several DT are constructed, each giving more weight to training samples that were wrongly classified in the previous DT. For every input case, the different DT vote for a certain target class. The input data is then assigned to the class with the most votes. From initial tests, and as confirmed by many studies [67-70], boosting significantly improved the performance of the DT. For each DT classification step, the number of boosts was set to ten.

### 3.5. Classification strategies

**Figure 3.**
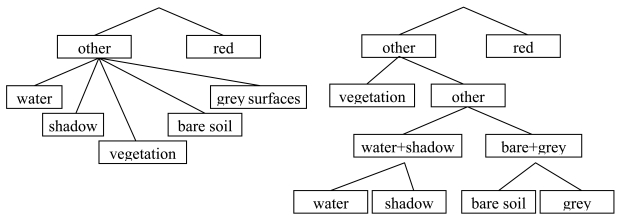
Classification strategies: two-step classification strategy (left) and full hierarchic classification strategy (right).

Both for DT and MLP classification, we compared two classification strategies: a two-step and a full hierarchic classification strategy. Because a separate segmentation was carried out for delineating red surfaces, this class was distinguished in a first classification step. Once red surfaces were identified, in the two-step classification strategy all image segments, obtained by re-segmenting the area not assigned to red surfaces, were assigned to one of the other classes, using one classification model (Figure 3, left). This strategy was also followed for the benchmark scenario using the nearest neighbour classifier.

In the full hierarchic strategy, classes were separated one by one from the remaining target classes, using different classification models. The order that was followed in this process was based on the complexity of the DT rules used for separating the classes. In every step all possibilities were tested: every class that still needed to be distinguished was separated from the remaining classes using a dedicated DT classification model. The DT obtained were mutually compared based on the number of input variables used and the complexity of the applied rules. The tree that required the least input variables and binary decision criteria was selected and the corresponding class was separated from the remaining classes using this classification model. This process was repeated until all classes had been distinguished (Figure 3, right). The same classification outline was followed for the full hierarchic MLP classification.

### 3.6. Shadow reclassification

Because of the strong variation in elevation in urban areas, many urban objects cast a shadow on their surroundings. Whatever the surface found underneath the shadow, all areas covered by shadow show similar spectral values [60]. In our classification scheme we therefore defined a separate class for shadow. To re-assign shadow areas to meaningful land-cover classes once the initial classification was performed, a new MLP classification was carried out. The land-cover classes present in the training objects that were labelled as shadow by the classifier – and that were known both for the training objects that actually belonged to other classes, as well as for the training objects that were labelled as shadow by the visual interpreter (the land-cover class underneath the shadows was also interpreted) – were used for training the MLP shadow reclassifier. As input for the training, the relative border length of the shadow object to neighbouring objects belonging to each of the other classes was used. As output the correct land-cover class was specified. By applying the MLP-model to all shadow objects, every object in the image is finally attributed to one of the target land-cover classes.

## 4. Results and Discussion

### 4.1. Feature selection

As explained above, five classification scenarios were tested. First the benchmark nearest neighbour classification was carried out. Second, both the DT and the MLP algorithm were applied following the two-step and the full hierarchic classification strategy. In every phase of the DT and the MLP classification a feature selection from the 29 input variables was carried out to separate the considered classes. Table 3 gives an overview of the selected variables for every phase in the two-step and in the full hierarchic classification approach, using both DT and MLP classification algorithms. The classification of red surfaces is the same for both approaches and the selected variables for this step are therefore only outlined in the table on the left.

As can be observed, almost all of the 29 input variables are used in one or more of the classification models developed. Only the entropy calculated on the green band and the angular second moment calculated on the red band are never selected. All the other texture variables derived from the Haralick co-occurrence matrix are used.

In the two-step classification strategy, the DT and the MLP classifiers make use of respectively 18 and 20 different input variables. In the full hierarchic classification strategy, the DT uses more different input variables (20), while the MLP uses less (15). The use of such a large number of input variables gives an indication of the difficulty of separating a limited number of land-cover classes within an urban setting using high spatial resolution data with a limited spectral resolution, like Ikonos imagery. It should be noted that the two algorithms select a different combination of input features. Also for the class-specific splits the selected variables often do not correspond.

### 4.2. Classification

Using the exhaustive visual interpretation of the three morphologically distinct ground truth sites, confusion matrices were generated for the five classification approaches. From these matrices, percentage correctly classified pixels (PCC), kappa indices and per class user's accuracies were derived (Table 4). For every approach PCC values are above 75%, while kappa values are between 0.69 and 0.77. The four scenarios using the more sophisticated learning algorithms that had the option to choose from the 29 selected input variables outperform the benchmark nearest neighbour classification that uses eight input variables with at least two percentage points. The nearest neighbour classifier performs substantially worse for classes that seem to be hard to distinguish, namely shadow and bare soil.

Comparing the different accuracies shows substantial differences between the classification results obtained for the different scenarios using the more sophisticated learning algorithms. In general, neural networks are expected to perform better than decision trees when trying to distinguish classes that are, given their spectral characteristics, difficult to separate. Nevertheless, with a difference of 2%, it is the DT that obtains a higher accuracy for the two-step classification approach. The accuracy of the DT classifier does not improve using the full hierarchic classification strategy. This may be explained by the characteristics of the DT algorithm, which is based on a step-by-step splitting of cases in subclasses, with specific target classes showing up as leaves in different sections of the tree. By creating extra splits in a later stage of the tree, the algorithm can optimally account for the presence of land-cover subclasses with distinct properties in splits initially defined too broadly. Applying a hierarchic, stepwise strategy, controlled by the image expert, each land-cover class is separated from the remaining target classes, without the possibility of separating other members of the class with different characteristics in a later stage of the classification. For the MLP approach however, applying the full hierarchic strategy substantially increases the accuracy of the classification, from a kappa value of 0.71 to a kappa value of 0.77. This can be explained by the fact that in the full hierarchic approach, each network gets a simpler problem to solve.

The user's accuracy for the grey surfaces class, which occupies one third of the validation area, has for every strategy a very high value (between 0.82 and 0.88). The user's accuracies for vegetation and water are above 0.93 and above 0.97 respectively for each scenario. For the red surfaces class, which is separated from the other classes in the first step of every classification approach, only the DT classification model was able to reach a high user's accuracy of 0.89. For the MLP approach a user's accuracy for red surfaces of only 0.76 was obtained. The MLP thus overestimates the amount of red surfaces to a higher degree than the DT model.

**Figure 4.**
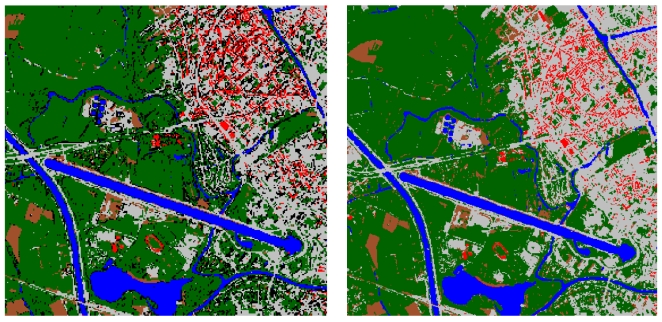
Full hierarchic multi-layer perceptron classification results: original classification (left) and result after post-classification shadow re-assignment (right).

The user's accuracies for the bare soil class are somewhat lower. A good distinction between bare soil and artificial surfaces is particularly important in the context of sealed surface mapping. It is therefore important to note that the user's accuracies increase from below 0.50 in the benchmark nearest neighbour classification, to above 0.60 for the DT and MLP classification scenarios. The use of additional variables and more sophisticated learning algorithms seems to be very helpful in separating this important target class from the other classes. Among the four scenarios based on DT and MLP, a clear difference can be noticed between the two-step and the full hierarchic strategy. Bare soil is clearly better classified when applying a full hierarchic strategy, where dedicated models are used to separate bare soil from the other classes. Besides the differences in performance between the two strategies, there is also a difference in performance between the two algorithms. Using a full hierarchic strategy, the MLP algorithm clearly produces higher accuracies than the DT algorithm for the mapping of bare soil (0.83 against 0.70 for DT). As stated above, neural networks are indeed expected to perform better than decision trees when trying to distinguish classes that are, given their spectral characteristics, difficult to separate. The hierarchic MLP classification is also the approach that generally performs best. The classification result obtained with this scenario is shown in figure 4 (left). The full confusion matrix for this scenario is given in table 5.

The confusion matrix for the full hierarchic MLP classification approach shows that not only the user's accuracy, but also the producer's accuracy (0.92) for bare soil is very high. Confusion with red and grey surfaces is limited, which is important for sealed surface mapping. Furthermore it is clear that water is most easily differentiated from other classes, with accuracies not below 0.91. For vegetation a producer's accuracy of 0.83 and a user's accuracy of 0.93 are obtained. The user's accuracy of the red surfaces class is only 0.76, yet most confusion is with grey surfaces. Since red surfaces and grey surfaces both constitute the sealed surface area, confusion between both classes poses no particular problem. More problematic is the relatively high proportion of grey surface objects that is attributed to the shadow class, leading to a producer's accuracy for grey surfaces of 0.78, and a user's accuracy for objects that are actually covered by shadow of only 0.30. The occurrence of shadow is thus largely overestimated due to confusion with dark grey surfaces. This problem was dealt with in the shadow reclassification phase, where pixels that are wrongly classified as shadow, as well as pixels that are actually covered by shadow, are re-assigned to one of the five land-cover classes. The shadow reclassification procedure was applied only to the classification result with the best overall classification accuracy, i.e. the full hierarchic MLP approach.

### 4.3. Shadow reclassification

In the hierarchic MLP classification result, the shadow class covers about 13% of the validation area. The objects classified as shadow conceal the underlying land-cover information in which the user of the land-cover map is interested. To uncover this information, a shadow reclassification using a new MLP was performed. As explained before, the MLP shadow reclassifier models the relationship between the relative length of the border which the shadow object shares with the neighbouring objects assigned to each of the classes, and the target land-cover class of the shadow object itself. By reassigning shadow patches to meaningful land-cover classes, the information content of the classified image is strongly increased.

Because of the low user's accuracy of the shadow class in the initial classification, the shadow reclassification substantially improves the overall accuracy of the land-cover map (Table 6). The PCC increases from 82.5% to 88.5%, the kappa index from 0.77 to 0.84. Figure 4 shows the result of the MLP full hierarchic classification for the entire study area, before and after shadow removal.

Confusion in the final classification result (Table 6) mostly occurs between red and grey surfaces, and between vegetation and grey surfaces. Accuracies of red surfaces are more affected by this confusion than accuracies of grey surfaces and vegetation, because of the abundance of the latter classes (each one third) in the area used for validation. The user's accuracy of red surfaces (0.75) is therefore somewhat lower. Joined together with grey surfaces in the targeted sealed surface class though, the user's accuracy for sealed surfaces is as high as 0.87 and the producer's accuracy as high as 0.88.

Through shadow reclassification, however, the accuracy of the bare soil class is reduced compared to the original classification (the user's accuracy from 0.83 to 0.70, the producer's accuracy from 0.92 to 0.74). This demonstrates that, although the shadow reclassification procedure improves the overall accuracy, it does assign part of the objects labelled as shadow in the original classification to the wrong land-cover classes, leading to a reduction of user accuracies for all classes except for the grey surfaces class, which is most prominently present in the areas where the majority of image objects classified as shadow are found (see Figure 4, left). The decrease in accuracy is the most pronounced for bare soil. Nevertheless the accuracy for the bare soil class, which only covers a relatively small part of the area, is still quite high, and the confusion with sealed surface types limited considering the difficulties in separating bare soil from sealed surfaces reported in other studies [30, 41, 71, 72].

Because of the problems involved in correctly identifying bare soil in urban areas and the mostly low coverage of this class, many studies on urban mapping do not consider bare soil as a separate class [73]. For example, in much of the work on sealed surface mapping researchers simply consider bare soil as part of the sealed surface class [61], arguing that bare soil in urban areas usually has a high degree of imperviousness. If a proper identification of bare soil is considered important the use of hyperspectral data is suggested [74-76]. This study, however, demonstrates that a non-parametric hierarchical classification approach based on MLP, and using spectral as well as textural features, may allow an accurate mapping of sealed surfaces in the rural-urban fringe, where distinguishing sealed surfaces from bare soil is essential, even with the limited number of spectral bands offered by the Ikonos sensor.

## 5. Conclusions

In this study, four object-oriented classification strategies for the mapping of sealed surfaces were tested on Ikonos data for part of the rural-urban fringe of the city of Ghent (Belgium). A comparison was made between a full hierarchic classification approach, where classes are separated one by one from the remaining target classes, using a dedicated one-against-all classifier in each step, and a standard approach where one classification model is used for separating all classes. Both approaches were tested using a decision tree and a multi-layer perceptron classifier. For each classification problem, optimal input variables were selected out of a set of 29 spectral and texture features, calculated at the level of the image objects obtained after image segmentation. A benchmark nearest neighbour classification was carried out using only the four multispectral bands and the standard deviations on these bands as input. Each classification approach was exhaustively validated on a test data set, comprising of a high-density built-up area, a less dense urban area located at the urban fringe, and a rural site.

The four scenarios using the more sophisticated learning algorithms and an optimal subset of the 29 available classification features, outperformed the benchmark nearest neighbour classification using 8 input variables. Best results were obtained with the multi-layer perceptron classifier using a full hierarchic classification strategy which, compared to a standard non-hierarchic classification scenario, improved the overall accuracy from 78.2% to 82.5%. Accuracies obtained with the decision tree classifier, using a standard non-hierarchical approach were somewhat lower (80%) and could not be improved by applying a one-against-all hierarchic classification strategy.

An often reported problem in the mapping of sealed surfaces, using high spatial resolution data with a limited spectral resolution, is the confusion between bare soil and built-up surface types. While in the benchmark scenario a user's accuracy for bare soil of only 0.45 was reached, the use of a full hierarchic MLP approach improved the user's accuracy for bare soil to 0.83.

A simple context-based post-classification procedure was proposed for reassigning shadow objects in the land-cover map to the actual land-cover type present beneath the shadow, using information on the relative length of the border shared with neighbouring objects belonging to the other classes. By reassigning each shadow object to one of the target land-cover classes, both the information content and the overall accuracy of the final land-cover map could be substantially improved. An increase of the PCC with 6 percentage points and a kappa of 0.84 were obtained. Sealed surfaces are accurately mapped with values of 0.87 and 0.88 for the user's and producer's accuracy.

## Figures and Tables

**Table 1.** Total number of validation pixels for each target class, relative weight of each class in the validation set, and colour used for representing each class in the maps.

	**red surfaces**	**vegetation**	**water**	**bare soil**	**grey surfaces**	**shadow**	**total**
**total**	74,868	436,972	195,490	47,842	440,182	68,436	1,263,790
	*5.9%*	*34.6%*	*15.5%*	*3.8%*	*34.8%*	*5.4%*	*100%*
	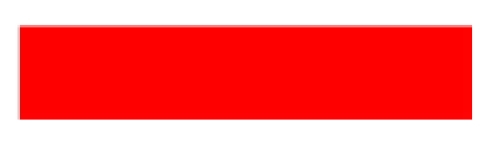	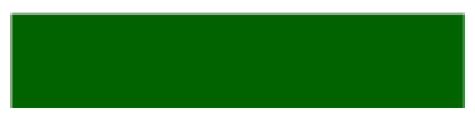	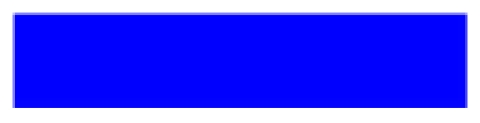	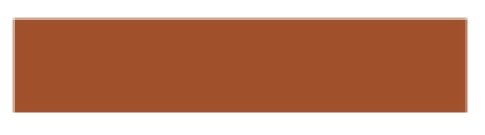	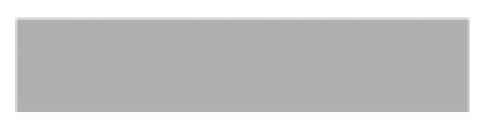	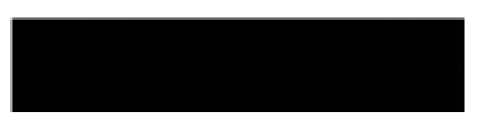	

**Table 2.** Overview of the 29 spectral and textural variables used as input for the variable selection in each step of the DT and MLP classification scenarios.

**mean (x4)**	average of the values in the spectral band, taken over all pixels within the segment
**ratio (x4)**	ratio between the mean of the spectral band and the sum of the mean values in every spectral band within the segment
**stddev (x4)**	standard deviation of all pixel values in the spectral band within the segment
**glcm asm (x4)**	angular second moment of the grey-level co-occurrence matrix: reflects the degree of homogeneity present in the spectral band within the segment
**glcm contrast (x4)**	contrast of the grey-level co-occurrence matrix: reflects the contrasts present in the spectral band within the segment
**glcm entropy (x4)**	entropy of the grey-level co-occurrence matrix: reflects the randomness in the spatial arrangement of spectral band values within the segment
**ratio green-blue**	average of the green band divided by average of the blue band
**ratio red-blue**	average of the red band divided by average of the blue band
**ratio red-green**	average of the red band divided by average of the green band
**brightness**	sum of the mean values in every spectral band
**ndvi**	(nir - red) / (nir + red)

**Table 3.** Selected input variables for each phase in the two-step and the full hierarchic classification strategy for DT and MLP classification. In the two-step strategy, ‘red’ stands for the red surfaces class and ‘rest’ for the other classes, which are classified in one step in this approach.

	**two-step strategy**	
	**DT**	**MLP**
**red**	mean nir ratio red-green	mean green ratio nir ndvi
**rest**	mean blue stddev blue mean green ratio green stddev green glcm asm green stddev red glcm contrast red mean nir stddev nir asm nir ratio green-blue ratio red-blue ratio red-green brightness ndvi	ratio blue stddev blue glcm asm blue glcm entropy blue mean green ratio green glcm asm green glcm contrast green mean red ratio red stddev red mean nir ratio nir glcm contrast nir ratio green-blue ratio red-blue brightness

	**full hierarchic strategy**	
	**DT**	**MLP**
**vegetation**	mean blue mean red ratio red ratio nir stddev nir ratio red-green	stddev red glcm contrast red ratio nir glcm entropy nir ratio red-blue ratio red-green
**water+shade <> bare+grey**	stddev blue mean green ratio green stddev red mean nir ratio green-blue ratio red-green	mean green ratio green mean red stddev red contrast nir
**water<>shade**	glcm asm green glcm entropy red glcm asm nir ratio green-blue brightness	mean blue ratio blue ratio green-blue
**bare<>grey**	ratio blue stddev blue glcm contrast blue ratio green stddev green ratio red-blue	mean blue mean red stddev red glcm contrast nir glcm entropy nir

**Table 4.** Per class user's accuracies, PCC and kappa values for the five classification scenarios.

		**nearest neighbour**	**two-steps**		**hierarchic**	

			**DT**	**MLP**	**DT**	**MLP**

**user's accuracy**	**red surfaces**	0.79	0.89	0.76	0.89	0.76
	**vegetation**	0.95	0.93	0.93	0.94	0.93
	**water**	0.97	0.99	0.97	0.99	0.98
	**bare soil**	0.46	0.60	0.65	0.70	0.83
	**grey surfaces**	0.82	0.86	0.86	0.84	0.88
	**shadow**	0.19	0.27	0.25	0.26	0.30

**PCC**		76.2%	80.4%	78.2%	79.3%	82.5%
**kappa**		0.69	0.74	0.71	0.73	0.77

**Table 5.** Confusion matrix for the full hierarchic multi-layer perceptron classification result before shadow reclassification.

		**exhaustive validation**						**sum**	**user's**
		**red**	**veg**	**water**	**bare**	**grey**	**shadow**		**accuracy**
**classification**	**red**	**64763**	221	464	25	17985	1892	85350	0.76
	**veg**	282	**362442**	5458	2167	11834	5951	388134	0.93
	**water**	0	2539	**177895**	0	323	615	181372	0.98
	**bare**	1375	2305	757	**43838**	4482	209	52966	0.83
	**grey**	4386	31051	1672	736	**343980**	10114	391939	0.88
	**shadow**	4062	38414	9244	1076	61578	**49655**	164029	0.30
**sum**		74868	436972	195490	47842	440182	68436	1263790	
**producer's accuracy**		0.87	0.83	0.91	0.92	0.78	0.73	PCC: 82.5% kappa: 0.77	

**Table 6.** Confusion matrix for the full hierarchic multi-layer perceptron classification result after shadow reclassification.

		**exhaustive validation**					**sum**	**user's accuracy**
		**red**	**veg**	**water**	**bare**	**grey**		
**classification**	**red**	**64048**	228	482	26	20343	85127	0.75
	**veg**	1520	**392315**	7198	9655	23470	434158	0.9
	**water**	2	4388	**184960**	1018	1287	191655	0.97
	**bare**	1412	7597	1009	**51436**	11826	73280	0.7
	**grey**	7886	36571	1841	7748	**425524**	479570	0.89
**sum**		74868	441099	195490	69883	482450	1263790	
**producer's accuracy**		0.86	0.89	0.95	0.74	0.88	PCC: 88.5% kappa: 0.84	
